# Nonlinear Interactions and Spatial Heterogeneity of Ecosystem Service Transitions in an Ecologically Fragile Karst Region of Southwest China

**DOI:** 10.1002/ece3.72401

**Published:** 2025-12-11

**Authors:** Yangyang Wu, Jinli Yang, Siliang Li, Yongli Mu, Dokrak Marod, Chunzi Guo, Xiaodong Yang, Huancai Feng, Runze Wu, Haobiao Wu, Panli Yuan, Shaopeng Feng, Songtao Li, Xuegang Xing, Guangjie Luo

**Affiliations:** ^1^ Guizhou Provincial Key Laboratory of Geographic State Monitoring of Watershed Guizhou Education University Guiyang China; ^2^ Department of Life Sciences Imperial College London London UK; ^3^ School of Earth System Science Tianjin University Tianjin China; ^4^ College of Ecology and Environment Xinjiang University Urumqi China; ^5^ Department of Forest Biology, Faculty of Forestry Kasetsart University Bangkok Thailand; ^6^ Administration of Ecology and Environment of Haihe River Basin and Beihai Sea Area Ministry of Ecology and Environment of People's Republic of China Tianjin China; ^7^ Department of Geography and Spatial Information Techniques Ningbo University Ningbo China

**Keywords:** ecological sensitivity, ecosystem service value, geodetector, karst watershed, land use change

## Abstract

Karst regions are ecologically fragile landscapes where land use transitions and human interventions significantly reshape ecosystem service value (ESV). This study investigates the Miaoling watershed in Southwest China—a pivotal ecological barrier—by integrating a value equivalent factor model with an optimized geographic detector to quantify ESV changes and their drivers from 2000 to 2020. Our results reveal substantial land conversions, particularly between forest, grassland, and cropland, leading to pronounced spatial heterogeneity in ESV. Mixed‐use zones emerge as critical ecological bottlenecks where gains in ESV coincide with heightened environmental risks. Importantly, we uncover strong nonlinear interactions between climatic (NDVI, precipitation) and socioeconomic (primary industry output) factors, reinforcing the complexity of natural–human feedbacks in karst landscapes. This study offers new insights into the spatial targeting of ecological restoration and risk management and contributes a replicable framework for assessing ecosystem transitions in vulnerable environments.

## Graphical abstract

We investigated the spatiotemporal evolution and driving mechanisms of ecosystem service value (ESV) in the ecologically fragile Miaoling karst region of Southwest China from 2000 to 2020. Our findings reveal that while vegetation restoration policies have enhanced ESV overall, spatial mismatches and ecological risks persist in mixed land‐use transition zones due to complex interactions between natural constraints and human activities. The study provides a replicable framework for ESV assessment and offers policy insights for land use regulation and ecological protection in mountainous regions.

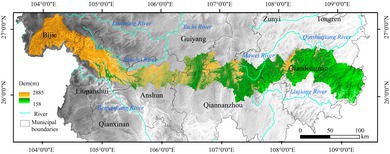

## 1 Introduction

Ecosystem service value (ESV), defined as the total value of direct and indirect benefits provided by natural ecosystems to human society, has become a critical indicator for guiding sustainable development strategies (Cusens et al. 2024). Land use change—particularly shifts in land use types and surface characteristics—directly influences the supply and sustainability of ecosystem services (Xu et al. 2024; Berhanu et al. 2023). Simulating and evaluating the effects of land use dynamics on ESV under varying scenarios not only helps quantify regional natural capital but also facilitates integrated ecological and economic management (Anley et al. 2022; Admasu et al. 2023). Therefore, a deeper understanding of how land use change shapes ESV is essential for developing effective conservation strategies and informing regional to global sustainability policies.

At the end of the last century, Costanza et al. (1997) pioneered a global assessment of grassland ESV, laying a foundational framework for valuing ecosystem functions. Building on this, Xie et al. (2015) proposed an ESV evaluation system tailored to China's socio‐ecological conditions, which has since been widely adopted in domestic ecological valuation research. Subsequent studies have demonstrated that ecosystem services and their values are significantly influenced by climate variability and anthropogenic disturbances—particularly land use change (Anley et al. 2022; Yuan et al. 2022). Recent research has shifted from static valuation toward dynamic assessments that incorporate both temporal and spatial heterogeneity of ESV (Berhanu et al. 2023). Multi‐scale analyses have become increasingly common, linking landscape fragmentation, vegetation productivity, and socio‐economic drivers to ESV change (Ziaul Hoque et al. 2022). Meanwhile, empirical studies have uncovered trade‐offs and synergies among different service categories (e.g., provisioning vs. regulating services), especially in regions undergoing rapid land cover transitions (Anley et al. 2022). With the advancement of geospatial technologies, remote sensing and GIS have enabled more refined monitoring and modeling of ESV spatiotemporal dynamics (Ziaul Hoque et al. 2022). These tools are particularly effective in ecologically fragile regions, such as the karst landscapes of Southwest China, where rapid land use shifts and topographic complexity pose challenges for traditional ecological monitoring (Yuan et al. 2022).

The karst regions of Southwest China deliver a wide array of critical ecosystem services, including water source protection, soil conservation, and carbon sequestration. These services are foundational to maintaining ecological balance and supporting sustainable socio‐economic development across the Yangtze and Pearl River basins (Li et al. 2021; Chen et al. 2021). However, the region is characterized by a pronounced human–land conflict, where fragile geological conditions and intense anthropogenic pressures result in frequent and large‐scale land use changes. Climate change and human activity jointly exert profound impacts on ecosystem structure, function, and service provision, thereby posing long‐term challenges to ecological security in karst landscapes (Wu et al. 2023; Pu et al. 2022). Regional studies of Sichuan and Guizhou have highlighted that shifts in forest, grassland, and cropland areas are major drivers of ESV changes, with forest expansion being particularly beneficial for enhancing ESV (Li and Luo 2023). Despite this progress, much of the existing literature adopts a linear framework that traces land use change to ESV response (Zhang et al. 2022; Wu and Wang 2024), while neglecting the underlying ecological processes and feedback mechanisms. Moreover, few studies have systematically explored the spatiotemporal variability of ESV and its multifactorial drivers within ecologically sensitive transition zones such as river basin watersheds.

As a critical watershed in the Southwest Karst region that separates the Yangtze and Pearl River basins, the Miaoling Range encompasses key ecological functional zones such as the Miaoling National Forest Park, Caohai National Nature Reserve, and Leigongshan National Nature Reserve. These protected areas play a pivotal role in sustaining regional ecosystem service capacity and ecological stability (Wu et al. 2023). Land use change has been identified as the primary factor influencing variations in the Normalized Difference Vegetation Index (NDVI) across the region. Since 2000, both NDVI and the ecological health index have exhibited a “V”‐shaped trajectory, with a notable recovery occurring after 2010 (Pu et al. 2022). However, the inherent ecological fragility of karst landforms intensifies the sensitivity of the environment to economic development and anthropogenic activities (Gong et al. 2022; Wu et al. 2022). In this context, a rigorous evaluation of the spatiotemporal heterogeneity and coupled interactions between land use change and ESV is essential for enhancing ecosystem service functions and achieving a coordinated ecological–economic development pathway. Such understanding holds practical significance for maintaining ecological balance and advancing sustainable development strategies in the Miaoling region.

In view of the above, this study adopts a widely applied value equivalent framework for ecosystem service assessment (Xie et al. 2015), in combination with an optimal parameter geodetector model (Wu et al. 2023). Focusing on the Miaoling region—a karst watershed that bridges the Yangtze and Pearl River basins—we integrate multi‐source datasets, including ecological remote sensing and socio‐economic data, to explore the following three scientific questions: (1) What are the characteristics of territorial spatial evolution and ecological sensitivity in the Miaoling region over the past 20 years? (2) How has the ESV varied spatiotemporally from 2000 to 2020? (3) What are the effects of multi‐dimensional driving factors on ESV patterns in the region?

This study conducts a systematic analysis of land use transitions, ecological risk distributions, ESV dynamics, and their underlying drivers in the Miaoling region. The findings are expected to support the development of targeted ecological protection strategies, inform land use planning and restoration initiatives, and contribute to the improvement of ecosystem service capacity and regional sustainability in karst watersheds.

## 2 Materials and Methods

### 2.1 Description of Study Area

**FIGURE 1:**
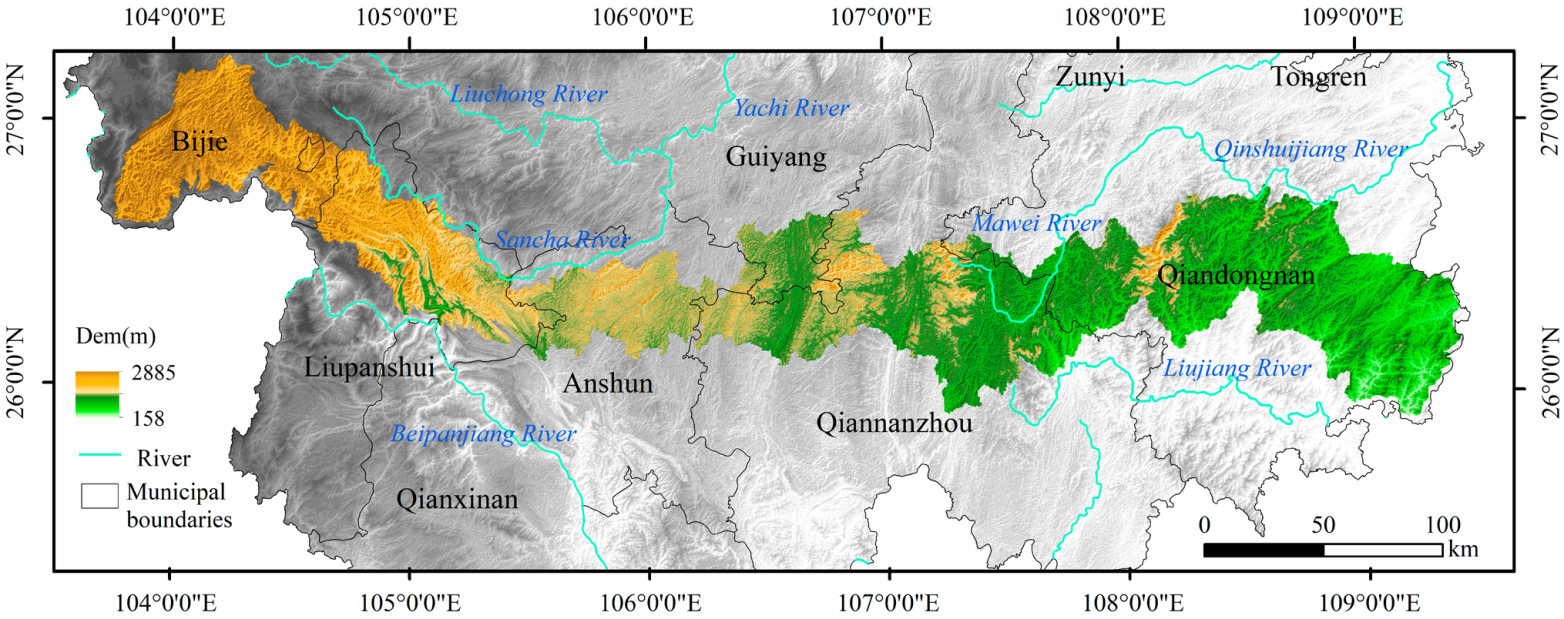
Map of the study area of Miaoling mountains.

Miaoling, situated in central Guizhou Province, China (25.73°–27.16° N, 103.82°–109.48° E), serves as a key watershed on the karst plateau separating the Yangtze and Pearl River basins (Figure 1). It is part of the “one range, eight basins” geomorphological system of the Southwest Karst Plateau, encompassing river basins such as Yuanjiang, Liujiang, Wujiang, Qijiang–Chishuihe, Hongshuihe, Niulanjiang–Henghe, Beipanjiang, and Nanpanjiang river basin (Wu et al. 2023). The region has a highly varied topography (158–2885 m) and a warm, humid climate with a mean annual temperature of ~15.5°C and annual precipitation between 1000 and 1100 mm, providing favorable hydrothermal conditions for vegetation. Dominant vegetation types include mid‐subtropical evergreen broad‐leaved forests, mixed evergreen–deciduous forests, deciduous broad‐leaved forests, and shrub meadows. As one of Guizhou's major forested areas, Miaoling delivers essential ecosystem services, including biodiversity conservation, soil and water retention, and climate regulation, making it a critical ecological security barrier for the Southwest Karst region.

### 2.2 Data Sources and Processing

This study analyzed land use, ESV, and their driving factors in the Miaoling region for the years 2000, 2010, and 2020, utilizing multi‐source datasets (Table 1). Land use data encompassed six categories: forest, cropland, grassland, water bodies, built‐up land, and unused land. All spatial datasets, including NDVI, temperature, and precipitation, had a resolution of 30 m to ensure consistency in analysis. In addition, vapor pressure deficit (VPD) and soil moisture (SM) data with a spatial resolution of 1 km were incorporated. Socioeconomic data included the added value of Guizhou Province's primary, secondary, and tertiary industries for each study year, along with grain and fertilizer prices used in ESV estimation. Data processing, statistical analysis, and mapping were conducted primarily using R 4.4.1 and ArcMap 10.8.

**TABLE 1:** Data and sources.

Data name	Data source
DEM (Digital Elevation Model)	Geospatial Data Cloud (https://www.gscloud.cn/)
Average Unit Grain Yield	Guizhou Provincial Statistical Yearbook (https://www.guizhou.gov.cn/zwgk/zfsj/tjnj/)
Added Values of Primary, Secondary, and Tertiary Industries	
Average Grain Price	National Compilation of Agricultural Product Cost and Benefit Data (https://www.zgtjnj.org/)
Average Fertilizer Price	China Agricultural Information Network (http://www.agri.cn/)
Temperature and Precipitation Data	China National Earth System Science Data Center (https://www.geodata.cn/)
VPD (Vapor Pressure Deficit)	Climate Data Guide, National Center for Atmospheric Research (https://climatedataguide.ucar.edu/)
NDVI (Normalized Difference Vegetation Index)	Resources and Environment Science Data Center, Chinese Academy of Sciences (https://www.resdc.cn/)
Land Use Types	

### 2.3 Assessment of Land Use Transitions and Ecological Sensitivity

To comprehensively examine territorial spatial changes in the Miaoling region, this study applied the land use transfer matrix method to analyze the spatiotemporal evolution of land use patterns from 2000 to 2020. The conversion formula is as follows:

(1) $$S_{\mathrm{ij}}=\left[\begin{array}{cccc} S_{11} & S_{12} & \ldots & S_{1n}\\ S_{21} & S_{22} & \ldots & S_{2n}\\ \ldots & \ldots & \ldots & \ldots \\ S_{n1} & S_{n2} & \ldots & S_{\mathrm{nn}} \end{array}\right]$$

where $S_{\mathrm{ij}}$ the land use status at the beginning and end of the study period, and n denotes the number of land use categories. This analysis provides a robust foundation for evaluating the impacts of land use changes on ESV.

Ecological sensitivity is defined as the capacity of an ecosystem to adapt to external disturbances and recover its structure and function (Tong et al. 2018). Considering the regional geographic environment and human activity impacts, ten typical factors were selected for the ecological sensitivity assessment (Table 2). Among these, altitude, slope, aspect, soil texture, precipitation erosivity, VPD, and NDVI reflect the natural environmental sensitivity, while land use type, population density, and GDP indicate anthropogenic impacts.

**TABLE 2:** Single factor sensitivity grading standards.

Factor name	Sensitivity level				
	Insensitive	Mildly sensitive	Moderately sensitive	Highly sensitive	Extremely sensitive
Altitude (m)	< 1000	1000–1500	1501–2000	2001–2500	≥ 2501
Slope (°)	< 16	16–32	33–48	49–64	≥ 65
Aspect	Flat, South	SE, SW	East, West	NE, NW	North
NDVI	< 0.6	0.6–0.7	0.7–0.8	0.8–0.9	≥ 0.9
Distance to rivers (m)	> 10,000	800–10,000	500–800	200–500	< 200
Land use type	Impervious surface	Snow/ice, bare land	Farmland, shrubland	Grassland, wetland	Forest, water
Population density (persons km^−2^)	< 80	80–160	161–240	241–320	≥ 321
Soil texture	Sandy loam	Loamy sand	Loam	Sandy clay	Clay
Precipitation erosivity (MJ mm ha^−1^ h^−1^ a^−1^)	< 62,000	62,000–72,000	72,001–82,000	82,001–92,000	≥ 92,001
VPD (kPa)	< 0.3	0.3–0.4	0.4–0.5	0.5–0.6	≥ 0.6
GDP	2800	5600	8400	11,200	14,000

Resistance analyses for the ten key factors were conducted using the Euclidean distance and kernel density methods. Subsequently, the Analytic Hierarchy Process (AHP) was employed to determine the relative weights of each factor (Table 3). The AHP consistency test yielded a maximum eigenvalue of 11.26 and a consistency index (CI) of 0.026, indicating that the consistency requirement was satisfied.

**TABLE 3:** Factor weights determined by AHP.

Factor name	Eigenvector	Weight value (%)
Altitude	0.443	4.029
Slope	0.428	3.894
Aspect	0.428	3.894
NDVI	0.666	6.058
Distance to rivers	0.666	6.058
Land use type	0.837	7.608
Population density	0.837	7.608
Soil texture	1.161	10.558
Precipitation erosivity	1.492	13.561
VPD	1.943	17.665
GDP	2.097	19.067

Finally, multi‐factor spatial overlay analysis was performed to map the spatial distribution of ecological sensitivity in the Miaoling region. The results were classified into five levels using the natural breakpoint method (Table 2), enabling precise identification of regional differences in ecological sensitivity.

### 2.4 Ecosystem Service Value Analysis

Based on the grain yield per unit area data provided in the Guizhou Provincial Statistical Yearbook (2020 Edition), this study adopted the revised equivalent table of ecosystem services per unit area in China as the foundational framework (Xie et al. 2015), and constructed a region‐specific equivalent factor table for ecosystem services in the Miaoling area (Table 4).

**TABLE 4:** Ecological service equivalent per unit of Miaoling area ecosystem.

Types of ecosystem services		Land use types					
		Crop	Forest	Grass	Water	Built‐up	Unused
Provisioning services	Food production	1.11	0.25	0.23	0.66	0.00	0.01
	Raw material production	0.25	0.58	0.34	0.37	0.00	0.03
	Water resource supply	−1.29	0.30	0.19	5.44	0.00	0.02
Regulating services	Gas regulation	0.89	1.91	1.21	1.34	0.00	0.11
	Climate regulation	0.47	5.71	3.19	2.95	0.00	0.10
	Environmental purification	0.14	1.67	1.05	4.58	0.00	0.31
	Hydrological regulation	1.50	3.74	2.34	63.1	0.00	0.21
Supporting services	Soil conservation	0.52	2.32	1.47	1.62	0.00	0.13
	Nutrient cycle maintenance	0.16	0.18	0.11	0.13	0.00	0.01
Cultural services	Biodiversity conservation	0.17	2.12	1.34	5.21	0.00	0.12
	Esthetic landscape	0.08	0.93	0.59	3.31	0.00	0.05

Meanwhile, considering the local socioeconomic development level, the economic contribution of grain output per unit area was adjusted accordingly. It assumes that, under natural conditions without human intervention, the economic value of one equivalent unit of ecosystem service is equal to one‐seventh of the economic value of grain production per unit area. The formula for calculating economic value is as follows:

(2) $$E_{a}=\frac{1}{7}\sum_{i=1}^{n}\frac{m_{i}p_{i}q_{i}}{M}$$

where $E_{a}$ is the economic value (¥/ha) of food production services provided by farmland ecosystems per unit area in the study region; *n* is the number of crop types; $p_{i}$ is the national average price (¥/t) of the *i*th crop in a certain year; $q_{i}$ is the yield per unit area (t/ha) of the *i*th crop in the study area; $m_{i}$ is the sown area (ha) of the *i*th crop in the study area; and *M* is the total planting area (ha) of all crops.

This study evaluated the ESV in Miaoling by aggregating four categories of services: provisioning, regulating, supporting, and cultural services (Guan et al. 2024). The equivalent factor method was employed for estimation, using the following formula:

(3) $$\mathrm{Es}=\mathrm{Ea}\times \sum_{i=1}^{7}e_{i}\times A_{i}$$

where Es (¥) is the total ESV of the study area; Ea (¥/ha) is the unit area economic value of farmland food production services; $e_{i}$ and $A_{i}$ denote the equivalent factor and land area for cropland, forest, shrubland, grassland, water bodies, bare land, and wetlands, respectively.

### 2.5 Optimal Parameter Geographic Detector

Spatial heterogeneity is a fundamental property of geographical phenomena. The geographic detector, a statistical method specifically designed to identify the driving forces behind spatial heterogeneity (Wu, Yang, et al. 2024), consists of four core modules: factor detection, interaction detection, risk zone detection, and ecological detection (Wu et al. 2023). To investigate the spatial relationship between ESV and its driving factors, this study employed an optimal parameter geographic detector model using the GD package in R. This approach enabled a quantitative analysis of the dominant drivers influencing spatial variation in ESV. The model is expressed as:

(4) $$q=1-\frac{\sum_{h=1}^{L}\;N_{h}\sigma_{h}^{2}}{N\sigma^{2}}=1-\frac{\mathrm{SSW}}{\mathrm{SST}}$$

(5) $$\mathrm{SSW}=\sum_{h=1}^{L}\;N_{h}\sigma_{h}^{2},\mathrm{SST}=N\sigma^{2}$$

where *h* = 1, 2,…, *L* is the stratification of the variable *Y* or the factor *X*; $N_{h}$ and N are the number of units in stratum *h* and the entire region, respectively; $\sigma_{h}^{2}$ and $\sigma^{2}$ are the variances of *Y*‐values in stratum *h* and the entire region, respectively; SSW and *SST* denotes the sum of variances within strata and the total variance of the entire region, respectively. The value range of *q* is [0, 1]. A larger *q* indicates more significant spatial heterogeneity in *Y*. If the stratification is generated by the independent variable *X*, a larger *q* signifies a stronger explanatory power of *X* for the attribute *Y*, and vice versa.

## 3 Results

### 3.1 Land Use Transitions and Spatial Patterns in Miaoling

From 2000 to 2020, land use types in the Miaoling region experienced notable changes (Table 5). Overall, forest area increased substantially between 2000 and 2010, with a net gain of 3885.51 km^2^. However, this trend reversed between 2010 and 2020, when the forest area decreased by 3317.82 km^2^, reaching a total of 12,537.22 km^2^ by 2020.

**TABLE 5:** Changes in land use types in Miaoling area from 2000 to 2020 (km^2^).

Year	Land use types					
	Grass	Cultivated land	Construction land	Forest	Water	Unused land
2000	5329.84	6987.34	176.84	11969.54	54.22	7.10
2010	5029.64	3326.45	235.48	15855.05	62.98	3.73
2020	4605.57	6700.48	559.78	12537.22	105.10	3.86
2000–2010	−300.19	−3660.89	58.64	3885.51	8.76	−3.37
2010–2020	−424.08	3374.02	324.30	−3317.82	42.12	0.13
2000–2020	−724.27	−286.86	382.94	567.69	50.88	−3.23

Grassland area showed a continuous decline over the two decades, decreasing by 724.27 km^2^ from 2000 to 2020. Cultivated land area decreased sharply between 2000 and 2010, falling from 6987.34 to 3326.46 km^2^, a reduction of 3660.89 km^2^. However, it rebounded between 2010 and 2020, increasing by 3374.02 km^2^ to reach 6700.48 km^2^.

Construction land expanded significantly over the study period, with an overall increase of 382.94 km^2^ from 2000 to 2020, including a growth of 324.30 km^2^ from 2010 to 2020. It reflects ongoing urbanization and industrialization in the region. Water area increased slightly by 50.88 km^2^ during the two decades, while unused land decreased from 7.10 to 3.86 km^2^.

**FIGURE 2:**
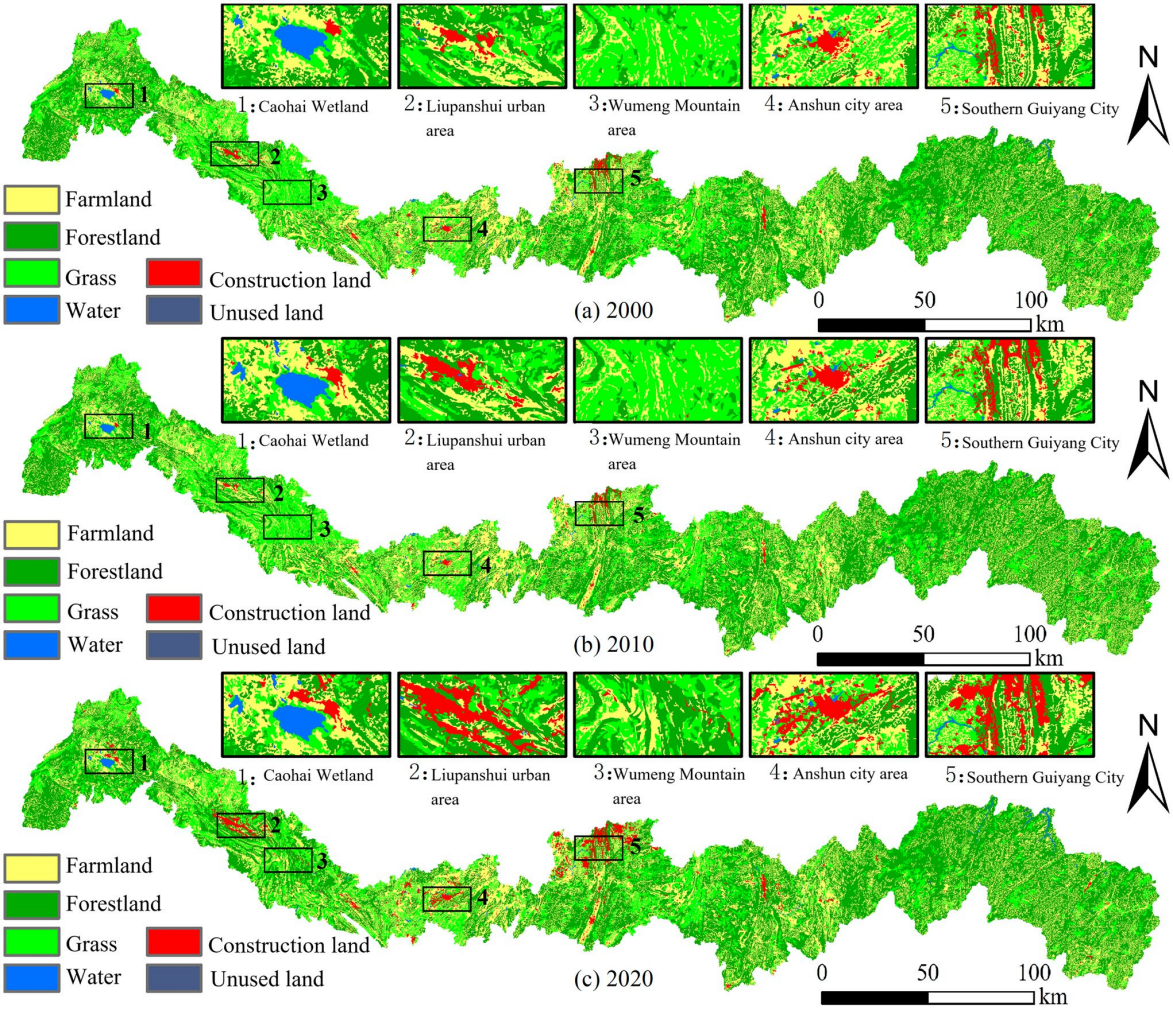
Spatial distribution of land use types in Miaoling area from 2000 to 2020.

Spatially, construction land exhibited marked expansion from 2000 to 2020, particularly around Guiyang City, Anshun City, Liupanshui City, and the Caohai region (Figure 2). Additionally, conversion of grassland to forest was evident in the Wumeng Mountain area of Liupanshui City.

### 3.2 Spatial Heterogeneity of Ecological Sensitivity in Miaoling

**FIGURE 3:**
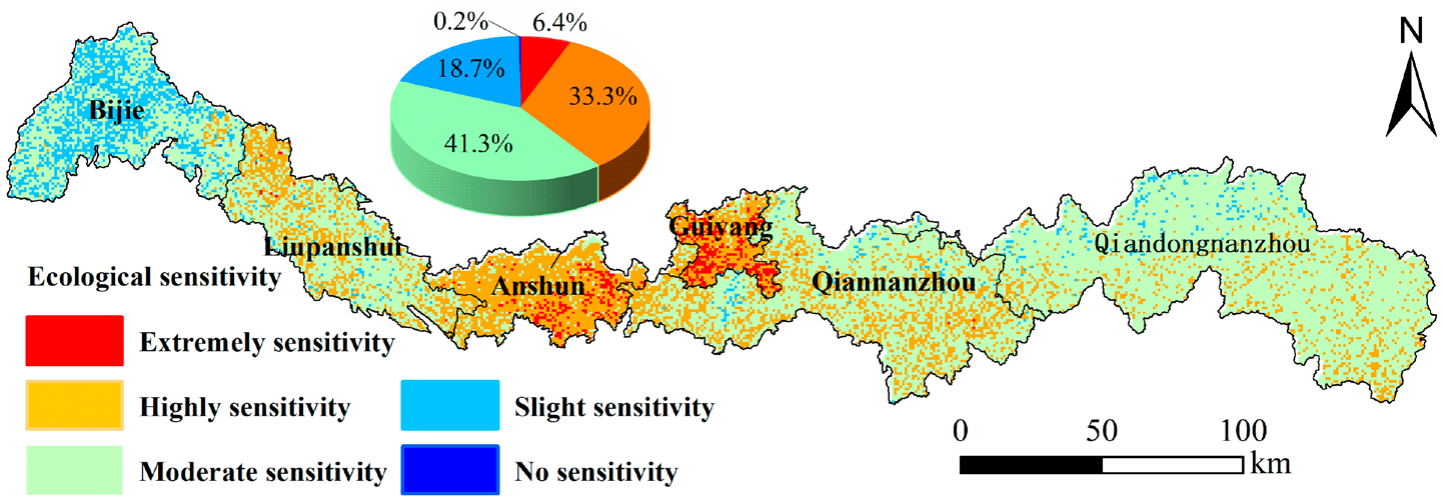
Spatial distribution characteristics of ecological sensitivity in the Miaoling area.

The results indicate marked spatial heterogeneity in ecological sensitivity across the Miaoling region. Areas classified as non‐sensitive, low sensitivity, moderate sensitivity, high sensitivity, and extreme sensitivity account for 0.2%, 18.7%, 41.3%, 33.3%, and 6.4% of the total area, respectively (Figure 3).

High‐ and extreme‐sensitivity zones are predominantly concentrated in Guiyang City and Anshun City (Guian New Area), where economic development is rapid. Significant high‐sensitivity areas are also distributed within Liupanshui City, Qiannan Prefecture, and Qiandongnan Prefecture. In contrast, non‐sensitive and low‐sensitivity areas are mainly found in Bijie City and parts of Qiandongnan Prefecture, while moderate‐sensitivity areas are widely distributed throughout the study region.

### 3.3 Spatiotemporal Dynamics of ESV in Miaoling

Between 2000 and 2020, the ESV in the Miaoling region generally exhibited an upward trend, increasing from ¥ 1.40 × 10^11^ to ¥ 1.72 × 10^11^, though significant regional differences were observed (Table 6). During this period, most areas recorded low average annual growth rates in ESV, with some even experiencing declines, reflecting marked spatial heterogeneity.

**TABLE 6:** The changes of ESV in Miaoling area (¥10,000).

Administrative area	ESV2000	2000–2010	ESV2010	2010–2020	ESV2020	2000–2020
Guiyang City	491940.71	−0.21%	490917.64	13.8%	558671.10	13.56%
Liupanshui City	1202724.47	0.39%	1207396.81	262.4%	4376809.88	263.9%
Anshun City	980072.28	0.61%	986043.61	103.0%	2002292.98	104.31%
Bijie City	1291037.87	0.42%	1296433.62	37.6%	1784989.05	38.2%
Qiandongnan Prefecture	6383420.10	0.73%	6430131.94	−15.2%	5451025.70	−14.6%
Qiannan Prefecture	3695961.21	0.35%	3708888.11	−18.1%	3036105.17	−17.83%
Total	14045156.63	0.53%	14119811.72	21.88%	17209893.87	22.53%

Specifically, Guiyang City's ESV decreased by 0.21% between 2000 and 2010, then increased by 13.8% from 2010 to 2020, resulting in an overall growth of 13.56%. Liupanshui City recorded a slight increase of 0.39% from 2000 to 2010, followed by a sharp rise of 262.4% between 2010 and 2020, culminating in an overall increase of 263.9%—the most substantial growth among all regions.

In Anshun City, ESV grew by 0.61% from 2000 to 2010 and by 103.0% from 2010 to 2020, yielding an overall increase of 104.31%. Bijie City's ESV rose by 0.42% in the first decade and by 37.6% in the second, with an overall growth of 38.2%.

Conversely, Qiandongnan Prefecture experienced an ESV increase of 0.73% between 2000 and 2010 but a decrease of 15.2% from 2010 to 2020, resulting in a net decline of 14.6% over the two decades. Similarly, Qiannan Prefecture saw an increase of 0.35% in the first decade, followed by an 18.1% decrease in the second, leading to an overall decline of 17.83%.

**FIGURE 4:**
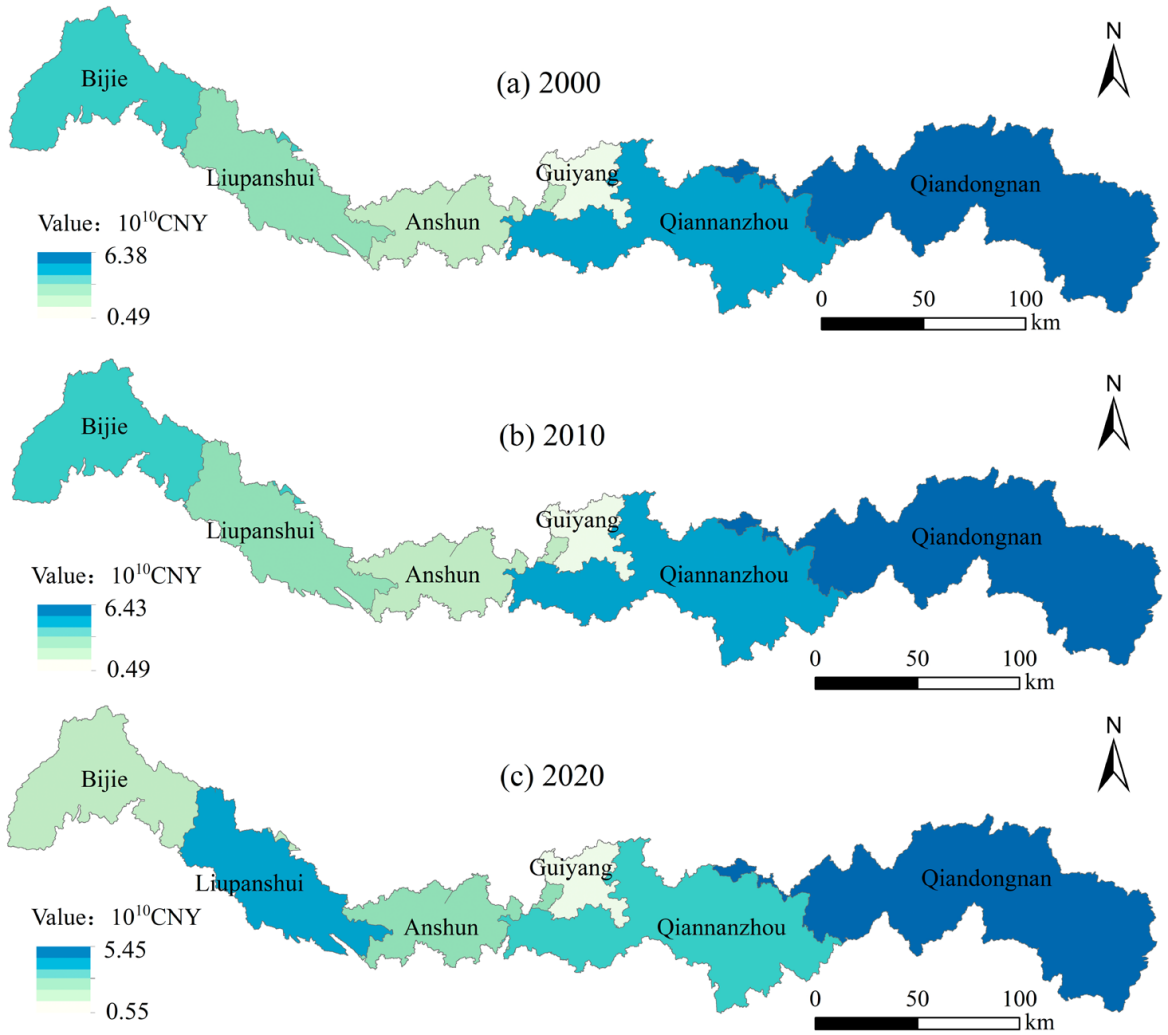
Spatial variation characteristics of ecosystem service value in Miaoling area.

Although Liupanshui City and Anshun City exhibited the most notable ESV growth, Qiandongnan Prefecture maintained the highest overall ESV in the Miaoling region throughout the study period (Figure 4).

### 3.4 Identifying Dominant Drivers of ESV Patterns

**FIGURE 5:**
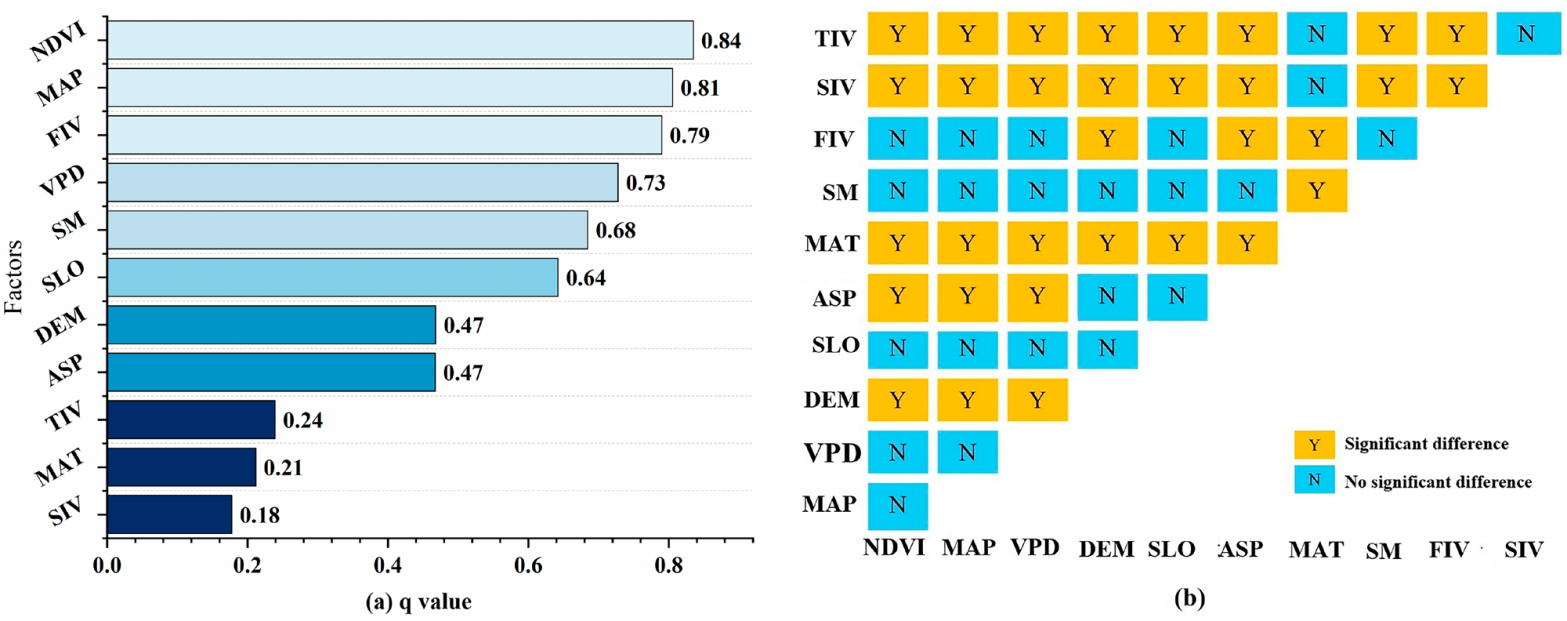
Single‐factor explanatory rate and significant differences of ecosystem service value in Miaoling area. ASP, aspect; DEM, digital elevation model; FIV, first industry value; MAP, mean annual precipitation; MAT, mean annual temperature; NDVI, Normalized Difference Vegetation Index; SIV, second industry value; SLO, slope; SM, soil moisture; TIV, third industry value; VPD, vapor pressure deficit.

The results indicate that the spatial heterogeneity of ESV in the Miaoling region is driven by multiple factors, ranked by their explanatory power (*q*‐values) from highest to lowest as follows: NDVI, precipitation, added value of primary industry, VPD, soil moisture, slope, elevation, aspect, added value of tertiary industry, air temperature, and added value of secondary industry (Figure 5).

Among these, NDVI (*q* = 0.84) and precipitation (*q* = 0.81) each explain over 80% of the spatial variation in ESV (Figure 5a), suggesting that climatic factors—particularly vegetation condition and precipitation—are the primary determinants of ESV distribution in the region. The added value of the primary industry (*q* = 0.79), representing socioeconomic drivers, also exerts a substantial influence on ESV spatial heterogeneity.

The impacts of these factors on ESV vary considerably (Figure 5b). Climatic variables, especially precipitation, exhibit significantly stronger explanatory power compared to topographic factors (elevation, slope, aspect) and socioeconomic factors such as the added value of the tertiary industry. In contrast, air temperature and the added values of the secondary and tertiary industries display limited influence on ESV distribution, with no significant differences observed among them. This further confirms that these factors contribute relatively less to ESV spatial variation compared to dominant climatic and primary industry drivers.

### 3.5 Nonlinear Interactions Among Drivers of ESV

**FIGURE 6:**
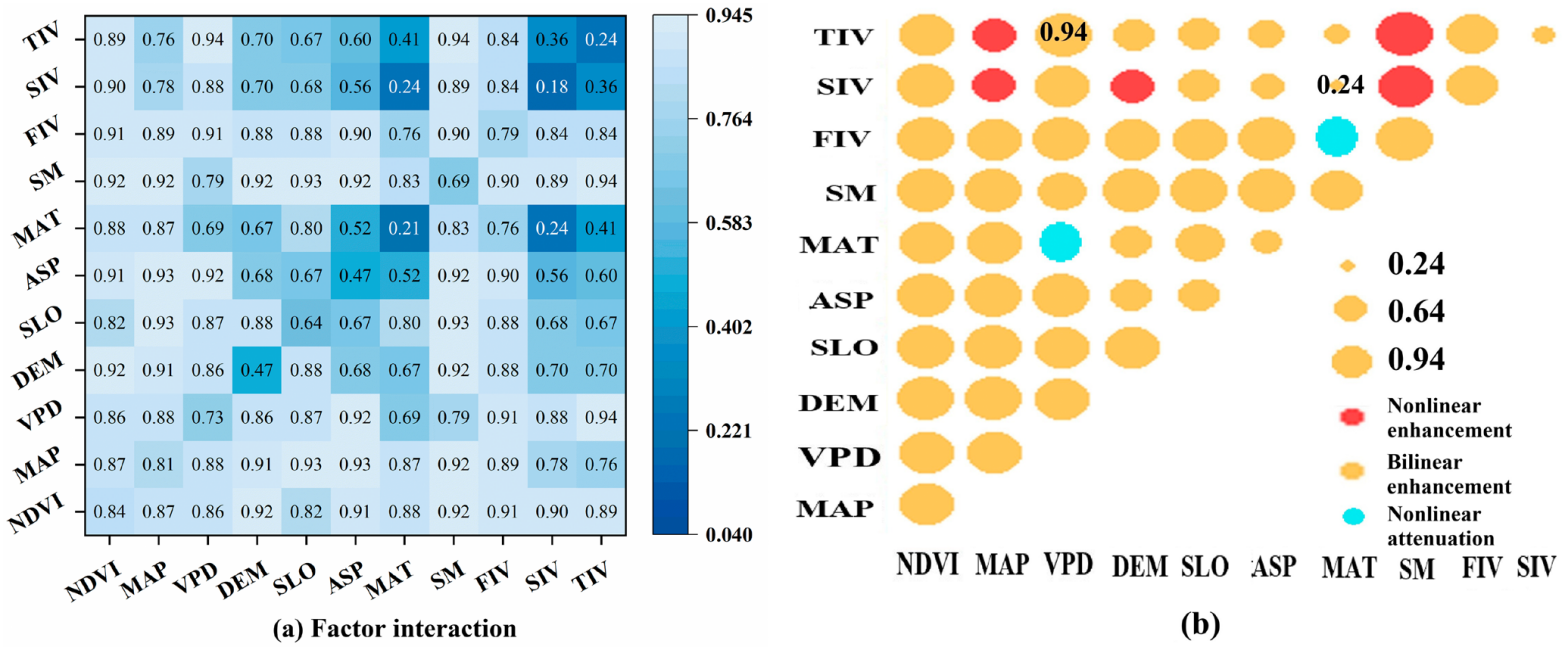
Interaction characteristics of driving factors on ecosystem service value in Miaoling area. The abbreviations are consistent with Figure 5.

Interaction detector analysis revealed that the spatial heterogeneity of ESV is primarily shaped by multi‐factor interactions. Two‐factor interaction *q*‐values were significantly higher than those of single factors, indicating a notable nonlinear enhancement effect (Figure 6).

Specifically, the interaction between NDVI and precipitation yielded a *q*‐value of 0.87, forming the strongest explanatory combination and confirming the decisive role of vegetation cover and moisture conditions in shaping ESV spatial patterns. The interaction between VPD and the added value of the tertiary industry showed an even higher *q*‐value of 0.94, revealing the nonlinear superimposition effect of climatic stress and economic development.

Interaction types exhibited significant differentiation. For example, the combination of NDVI and the added value of the primary industry (*q* = 0.83) demonstrated positive enhancement, reflecting the synergistic effects between ecological foundations and human activities. Conversely, the combination of air temperature and soil moisture (*q* = 0.21) exhibited negative weakening, suggesting that thermal conditions may attenuate hydrological regulation functions. Notably, the interaction between the added value of the secondary industry and air temperature produced the lowest *q*‐value (0.24) among all combinations, indicating spatial discontinuity between industrial activities and climatic factors.

Furthermore, climatic factors maintained high explanatory power at the regional scale, while topographic factors showed significant interaction enhancement (*q* > 0.8) in local karst landform areas, confirming the scale‐dependent characteristics identified by geographic detector analyses. These findings deepen the understanding of multi‐factor driving mechanisms underlying ESV spatial heterogeneity and provide a scientific basis for ecological conservation and sustainable development in karst regions.

## 4 Discussion

### 4.1 Topographic Constraints and Policy‐Induced Forest Recovery in Karst Urban Growth

Over the past two decades, the Miaoling region has experienced marked land use transitions, characterized by forest–cropland fluctuations, persistent grassland decline, and continuous expansion of built‐up areas. These shifts underscore the challenge of balancing ecological conservation with rapid urbanization and resource exploitation (Chen et al. 2023).

The conversion of rural land to urban and construction land, particularly around Guiyang, Anshun, Liupanshui, and the Caohai area, reflects a global trend in land transformation under development‐driven policies (Ziaul Hoque et al. 2022; Weiskopf et al. 2024; Qin and Sha 2023). This urban growth aligns with international patterns, underscoring the universality of urban spatial expansion under economic pressure (He et al. 2016). In Miaoling, urban growth is characterized by “strip‐shaped” expansion along mountain ranges in Liupanshui and Guiyang and “star‐shaped infilling” in Anshun (Figure 2), illustrating how karst terrain constrains urban spatial morphology (Wang, Liu, et al. 2022). These findings are consistent with observed urban expansion rhythms in Sichuan, Chongqing, China's coastal cities, and other global urbanizing regions (Chen et al. 2021; Yang et al. 2020; Rouet‐Leduc et al. 2024).

Since 2015, Guizhou Province has implemented comprehensive ecological protection and management plans focusing on restoring plateau ecological barriers, safeguarding river basin corridors, enhancing urban ecological quality, and maintaining biodiversity, supported by ten major ecological restoration projects (Wu et al. 2022; Guo et al. 2019; Wang, Zhang, and Chen 2022). The increase in forest area in Miaoling directly benefits from these initiatives, with grassland‐to‐forest conversion in the Wumeng Mountains exemplifying effective policy‐driven ecological restoration. National initiatives such as “Grain for Green” and ecological redline policies have played pivotal roles in protecting critical ecological regions across China, including the southwest karst area, Three‐North Shelterbelt, Qinghai‐Tibet Plateau, and Loess Plateau (Gong et al. 2022; Ma et al. 2023). These projects not only promote vegetation recovery and forest protection but also enhance ecosystem services and their economic value.

### 4.2 Balancing ESV Gains: Forest Stability vs. Mixed‐Use Fragility

Despite an overall upward trend in ESV, significant spatial heterogeneity persists in Miaoling. Highly sensitive areas are predominantly “forest–grassland–cropland mixed zones” which are hotspots for soil erosion and geological disasters, exhibiting pronounced ESV fluctuations (Wang et al. 2001). Forests provide crucial ecosystem services, including carbon sequestration and soil–water conservation, with their area changes directly influencing regional ESV dynamics. Qiandongnan Prefecture, with the densest forest cover, consistently maintains the highest ESV levels. Grassland‐to‐forest conversion in the Wumeng Mountains further illustrates forests' central role in sustaining ecosystem services (Ma et al. 2023; Zhou et al. 2021; Liu et al. 2023).

Given the high ecological sensitivity of “forest‐grassland‐cropland mixed zones” in the Wumeng Mountain area, Qiannan Prefecture, and eastern Qiandongnan (Figure 3), continuous ecological protection is essential to reduce ecological risks and maintain ESV growth. Non‐sensitive and low‐sensitivity areas are mainly in Bijie City and Qiandongnan Prefecture, while extremely sensitive areas cluster in Guiyang and Anshun, where urbanization intensity is highest. Divergent ESV trends across these regions reflect variations in development models and policy effectiveness (Gong et al. 2022; Ge et al. 2021).

Land use demands are driven by economic development and population growth, closely linked to the expansion of built‐up land and rapid industrial development. These changes often reduce biodiversity and degrade ecosystem services (Xiao et al. 2024; Aziz 2021), underscoring how forest protection, agricultural restructuring, urbanization, and industrialization policies shape regional ESV (Wu et al. 2025; Guo et al. 2018, 2023). Therefore, sustainable development in the Miaoling region hinges on adaptive, region‐specific land use strategies that balance ecological integrity with socioeconomic demands.

### 4.3 Water Stress and Human Drivers in Shaping Karst ESV Heterogeneity

ESV dynamics in Miaoling are closely linked to vegetation distribution, climatic conditions, ecosystem structure, and human activities (Rouet‐Leduc et al. 2024). In seasonally arid karst environments, increased precipitation enhances vegetation growth, while NDVI—a proxy for vegetation cover—strongly influences ecosystem functions (Wu et al. 2023, 2025; Hutyra et al. 2011). This study shows that climatic factors explain over 80% of ESV spatial heterogeneity, establishing them as primary drivers. By contrast, secondary and tertiary industry added values exhibit weaker explanatory power.

Notably, precipitation (*q* = 0.81) and VPD (*q* = 0.73)—key water stress indicators—exert greater impacts on ESV than air temperature (*q* = 0.21), emphasizing the dominance of water‐related factors in karst ecosystems. The strongest interaction effect (*q* = 0.94) occurred between VPD and tertiary industry added value, confirming that water stress and socioeconomic activities jointly shape ESV patterns, consistent with findings from Sichuan and the Loess Plateau (Feng et al. 2023; Ding et al. 2021).

Interaction detector results show that most factor combinations have higher explanatory power than single factors, with two‐factor and nonlinear enhancements dominating. High‐impact factors like NDVI and precipitation maintain strong explanatory power even when interacting with other variables. Socioeconomic–environmental factor interactions consistently enhance explanatory power, highlighting the importance of climate–society linkages in shaping ESV distributions. Conversely, interactions involving air temperature and factors such as VPD or secondary industry output exhibit nonlinear weakening effects, possibly due to temperature's context‐dependent impacts on ecosystem services in karst microclimates and its complex relationships with industrial activities (Wu et al. 2023; Wu, Wu, et al. 2024; Yang et al. 2024). The high topographic heterogeneity of karst landscapes also introduces spatiotemporal uncertainties in multi‐factor impacts, further complicating temperature's role in ESV dynamics.

## 5 Conclusions

This study presents a comprehensive assessment of the spatiotemporal evolution and driving mechanisms of ESV in the Miaoling karst mountainous region from 2000 to 2020. Despite an overall upward trend in total ESV, our findings reveal a coexistence of localized instability and spatial heterogeneity, reflecting the dual challenges of ecological fragility and intensified human–land conflicts.

ESV gains were primarily concentrated in areas with dense vegetation cover (NDVI > 0.8), particularly forest‐dominated zones. However, the transitional zones between forest, grassland, and cropland emerged as ecological bottlenecks—characterized by high sensitivity, elevated environmental risk, and unstable ESV dynamics. These zones function as interfaces where natural constraints (e.g., shallow soils, karst leakage) collide with socioeconomic pressures, particularly the expansion of the primary industry.

Crucially, our analysis reveals that nonlinear interactions between climatic (e.g., NDVI, MAP) and anthropogenic (e.g., land use change, primary industry output) drivers amplify ecological risks. For instance, the positive contributions of climate‐induced vegetation restoration are counteracted by land degradation from slope farming, triggering a mismatch between ESV growth areas and ecological risk zones.

To mitigate these risks and promote sustainable development, we propose a coordinated strategy that integrates natural resilience enhancement with socioeconomic regulation. Specifically, we recommend strict land use intensity thresholds (e.g., banning cultivation on slopes > 25°), the promotion of ecological agriculture, and the establishment of an NDVI‐based dynamic monitoring and early warning system. Moreover, a cross‐scale governance framework combining interface regulation, factor interaction optimization, and ecological compensation is essential for reconciling environmental protection with human development needs.

This study provides a spatially explicit framework and policy‐oriented insights for managing ecosystem service transitions in karst regions, offering a replicable reference for other ecologically fragile mountainous areas worldwide.

## Author Contributions

**Yangyang Wu:** conceptualization (equal), funding acquisition (equal), writing – original draft (equal). **Jinli Yang:** software (equal), visualization (equal), writing – original draft (equal). **Siliang Li:** conceptualization (equal), writing – review and editing (equal). **Yongli Mu:** formal analysis (equal), validation (equal). **Dokrak Marod:** methodology (equal), supervision (equal). **Chunzi Guo:** formal analysis (equal), writing – review and editing (equal). **Xiaodong Yang:** project administration (equal), supervision (equal), validation (equal). **Huancai Feng:** formal analysis (equal), validation (equal). **Runze Wu:** software (equal), visualization (equal). **Haobiao Wu:** data curation (equal), visualization (equal). **Panli Yuan:** data curation (equal), visualization (equal). **Shaopeng Feng:** data curation (equal), validation (equal). **Songtao Li:** funding acquisition (equal). **Xuegang Xing:** funding acquisition (equal). **Guangjie Luo:** funding acquisition (equal), resources (equal).

## Conflicts of Interest

The authors declare no conflicts of interest.

## Data Availability

All data supporting the findings of this study are openly available. Land use, NDVI, temperature, and precipitation data are archived and accessible from the China National Earth System Science Data Center (https://www.geodata.cn/). Digital elevation model (DEM) data are publicly available from the Geospatial Data Cloud (https://www.gscloud.cn/). NDVI time‐series data used for vegetation analysis are also available from the Resources and Environment Science Data Center of the Chinese Academy of Sciences (https://www.resdc.cn/). Socioeconomic data, including average grain yield and the added value of primary, secondary, and tertiary industries, are obtained from the Guizhou Provincial Statistical Yearbook (https://www.guizhou.gov.cn/zwgk/zfsj/tjnj/). Grain price data are sourced from the National Compilation of Agricultural Product Cost and Benefit Data (https://www.zgtjnj.org/), and fertilizer price data are provided by the China Agricultural Information Network (http://www.agri.cn/). Additionally, vapor pressure deficit (VPD) data are obtained from the Climate Data Guide of the National Center for Atmospheric Research (https://climatedataguide.ucar.edu/).
